# Patient satisfaction of primary care services in Gulf Cooperation Council Countries: A scoping review

**DOI:** 10.1002/jgf2.640

**Published:** 2023-07-12

**Authors:** Abdulaziz Alhenaidi, Waleed Al Nadabi, Asmaa Al‐Haqan, Hisham Kelender

**Affiliations:** ^1^ Directorate of Planning and Monitoring Ministry of Health Safat Kuwait; ^2^ School of Health and Wellbeing University of Glasgow Glasgow UK; ^3^ Ministry of Health Muscat Oman; ^4^ Department of Pharmacy Practice, College of Pharmacy Kuwait University Kuwait Kuwait

**Keywords:** GCC, Gulf Cooperation Council Countries, patient satisfaction, primary care, review

## Abstract

Patient satisfaction (PS) is an essential element in evaluating and improving healthcare systems. Few studies have gathered evidence about patient satisfaction with primary care (PC) services in the Gulf Cooperation Council (GCC) countries. Therefore, it is important to review the existing literature examining PS with PC services in the GCC countries. This scoping review was conducted for primary data studies published between 2000 to March 2022. Out of 4461 screened articles, 37 met the inclusion criteria. Most studies were conducted in Saudi Arabia and were of cross sectional methodology. PS with primary care in the GCC is affected by multiple provider‐related and patient‐related factors and users are generally satisfied with the service provided. Future research should focus on the effects of the factors that were not explored and validate the existing results.

## 1 BACKGROUND

The concept of Patient satisfaction (PS) within the scope of quality practices in health services has become a subject that has gained importance and has been studied extensively in recent years.^1^, ^2^, ^3^ The patient's thoughts and satisfaction with the service provided are essential in evaluating the service quality. PS can be defined as “the extent of agreement between what a patient expects to receive from the healthcare experience and the perception of the level of care they receive”.^4^ PS contributes to healthcare services' structure, process, and output.^5^

A valid way to learn patients' opinions about the health system is to systematically review the existing literature for studies that measure PS. With the increase in patient focus in the health field, there has been a significant increase in PS evaluation studies.^6^, ^7^ However, the scoping review of PS is still quite limited, specifically those conducted about PS with primary care (PC) services in the Gulf Cooperation Council (GCC) countries. A systematic review about PS in Saudi Arabia (one of the GCC countries) found that there was a contradiction between the patients' responses to the surveys on the domains of PS and their actual experience where patients reported that they were satisfied with primary care centers; however, they frequently attended the emergency department directly. The authors claimed that this indicated that patients were unlikely to be fully satisfied with the primary healthcare center services.^8^ A primary healthcare center in the GCC countries is a patient's first point of contact with the medical care system. The GCC countries share similar cultures and healthcare systems with some differences.

### 1.1 Objective

This scoping review aims to examine the nature and range of studies examining PS with PC services in the GCC countries. To the best of the researcher's knowledge, this is the first study that examines the nature and range of studies related to PS with PC services in the GCC countries.

## 2 METHODS

### 2.1 Review protocol

A scooping literature review was followed using Arksey and O'Malley framework^9^ to systematically summarize the nature and range of PS studies in the GCC countries in PC centers. It started with identifying the research question, then identifying relevant studies, and then went through the study selection process (Table 1).

**TABLE 1:** Application of Arksey and O'Malley framework.^9^

1. Identifying the research question: What is the existing evidence regarding PS of PC services in GCC countries. 2. Identifying relevant studies: Cumulative Index to Nursing and Allied Health Literature (CINHAL), EMBASE, MEDLINE (EBSCOhost, OVID, Web of Science), and PubMed were searched using the search terms in Table 1. 3. Study selection: inclusion and exclusion criteria in Table 2 4. Charting Data: included studies main information, title, publication date, authors, methodology, aims, and main outcome, were extracted in an excel sheet. A data extraction form was created, which includes the main information of each study included and satisfaction factors according to the systematic review by Batbaatar et. al.^10^ 5. Collating, Summarizing and Reporting the Results: Data was summarized numerically, which is the number of the studies in each of GCC countries, methodology and their focus; thematically were we used thematic analysis, the extracted data were charted and summarized according to the satisfaction factors by Batbaatar et. al.^10^

### 2.2 Search strategy

Search terms were initially used in Medline and applied to other databases. Field experts (clinicians and quality specialists) were consulted in the search terms selection process. Additionally, the terms used in other satisfaction‐related systematic reviews informed the search terms used in this review (Table 2). Terms appearing as keywords and subject headings were combined to search for studies that examined PS in primary health care in the Gulf region. The search was conducted in March 2022.

**TABLE 2:** Search terms.

Search terms for primary health care AND search terms for satisfaction AND search terms for users AND search terms for GCC countries			
Primary health care or PC or primary health center or PC center or primary health clinic or general practice or family medicine	Satisfaction or experience or views or perception or feedback	Users or patient or customers	Oman or Sultanate of Oman or Saudi Arabia or King Saudi Arabia or KSA or Kuwait or Bahrain or Qatar or United Arab Emirates or UAE *

*Note*: PC and satisfaction terms were searched separately with each country term.

### 2.3 Sources of data

Six databases (Table 1) were searched using the search terms. The papers included were empirical studies that examined PS with PC centers in any GCC countries published in peer review journals on or after the year 2000, whether in English or Arabic. Studies that examined satisfaction in hospital settings or satisfaction with specific clinical interventions or procedures were excluded (Table 3).

**TABLE 3:** Inclusion and exclusion criteria.

Inclusion	Exclusion
Studies exploring the PS with PC services English and Arabic Since the year 2000 Service improvement intervention (e.g., electronic records) Factors affecting PS Peer‐reviewed journal article Urban and rural centers	Before 2000 Non‐English or Arabic Non‐PC settings Specific clinical intervention or procedure Hospital outpatient settings Dental health services Telemedicine services Studies assessing the validity of survey instruments Commentary and non‐data studies Reviews and systematic reviews

### 2.4 Data extraction and data synthesis

Data were extracted and synthesized using tables to summarize and answer the review questions. The headings of tables summarizing the determinants of PS were developed following the systematic review by Batbaatar et al.^10^ The Preferred Reporting Items for Systematic Reviews and Meta‐Analyses (PRISMA) were used to guide the screening and selection process.^11^, ^12^ Title screening was conducted by one reviewer, while two reviewers conducted the abstract and full‐text screening. Any disagreement between the reviewers regarding the abstract screening was resolved by the third reviewer.

## 3 RESULTS

**FIGURE 1:**
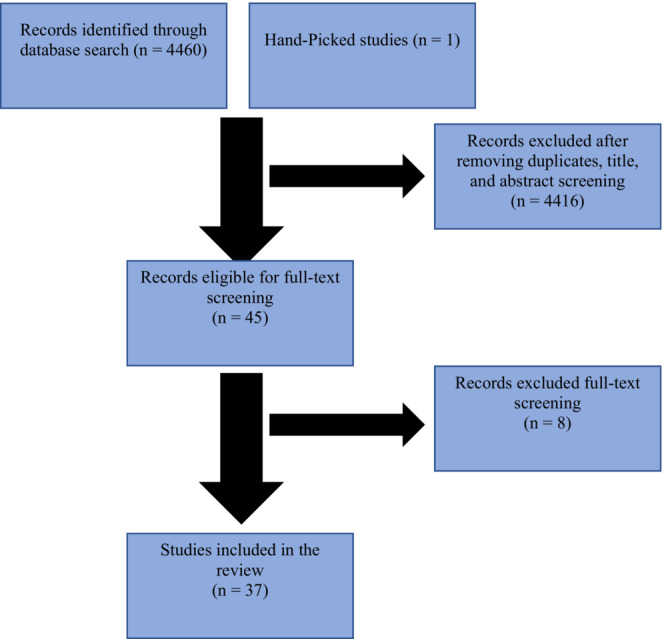
Flowchart of search and selection process using PRISMA.

The initial search identified 4461 studies; however, 37 were eligible for this scoping review (Figure 1, Table 4). Retrieved studies were from Bahrain (*N* = 1), Kuwait (*N* = 7), Oman (*n* = 6), Qatar (*N* = 2), and Saudi Arabia (*N* = 21). Almost all the studies were conducted using a cross sectional methodology. The themes identified in this review are presented according to the satisfaction determinants identified by Batbaatar et al.^10^ General satisfaction was an additional theme, and mostly it showed that participants were satisfied with the PC service provided in the Gulf region,^13^, ^14^, ^15^, ^16^, ^17^, ^18^, ^19^, ^20^, ^21^, ^22^, ^23^, ^24^, ^25^ with some studies cited moderate satisfaction,^21^, ^26^, ^27^, ^28^, ^29^, ^30^, ^31^, ^32^ and only a minority that they were dissatisfied.^33^, ^34^

**TABLE 4:** Included studies.

Reference	Country	Study type	Year of publication
Al‐Doghaither et al., 2000^29^	Kuwait	Cross sectional	2000
Al‐Doghaither and Saeed, 2000^31^	Saudi Arabia	Cross sectional	2000
Al‐Doghaither et al., 2001^41^	Kuwait	Cross sectional	2001
Saeed et al., 2001^30^	Saudi Arabia	Cross sectional	2001
Al‐Mandhari et al., 2004^46^	Oman	Cross sectional	2004
Mahfouz et al., 2004^22^	Saudi Arabia	Cross sectional	2004
Mohammed et al., 2004^19^	Qatar	Cross sectional	2004
Abdalla et al., 2005^26^	Saudi Arabia	Cross sectional	2005
Al‐Azmi et al., 2006^21^	Kuwait	Cross sectional	2006
Al Shetti and Al Sayyad, 2006^32^	Bahrain	Cross sectional	2006
Al‐Haqwi and Al‐Shehri, 2007^49^	Saudi Arabia	Cross sectional	2007
Al Dousari et al., 2008^37^	Kuwait	Cross sectional	2008
Al‐Sakkak et al., 2008^27^	Saudi Arabia	Cross sectional	2008
Al‐Azri and Ganguly, 2009^51^	Oman	Cross sectional	2009
Alhashem et al., 2011^39^	Kuwait	Cross sectional	2011
Albalushi et al., 2012^14^	Oman	Cross sectional	2012
Al‐Jafar, 2013^48^	Kuwait	Cross sectional	2013
Al‐Azri et al., 2014^35^	Oman	Cross sectional	2014
Al Shahrani and Baraja, 2014^17^	Saudi Arabia	Cross sectional	2014
Alotaibi et al., 2015^18^	Kuwait	Cross sectional	2015
Mohamed et al., 2015^25^	Saudi Arabia	Cross sectional	2015
Al Ali and Elzubair, 2016^38^	Saudi Arabia	Cross sectional	2016
Alfaqeeh et al., 2017^13^	Saudi Arabia	Cross sectional	2017
Almutairi, 2017^24^	Saudi Arabia	Cross sectional	2017
Bawakid et al., 2017^28^	Saudi Arabia	Cross sectional	2017
Tabekhan et al., 2018^33^	Saudi Arabia	Cross sectional	2018
Al Hasni and Al‐Rawajfah, 2019^16^	Oman	A pretest/posttest quasi‐experimental design was used.	2019
Al‐Kindi et al., 2019^20^	Oman	Cross sectional	2019
Alsayali et al., 2019^50^	Saudi Arabia	Cross sectional	2019
Alrasheedi et al., 2019^45^	Saudi Arabia	Cross sectional	2019
Elagi et al., 2019^23^	Saudi Arabia	Cross sectional	2019
Howsawi et al., 2020^44^	Saudi Arabia	Cross sectional	2020
Senitan and Gillespie, 2020^42^	Saudi Arabia	Cross sectional	2020
Wali et al., 2020^47^	Saudi Arabia	Cross sectional	2020
Alrabaie and Alzaydi, 2021^34^	Saudi Arabia	Cross sectional	2021
Al Zaidan et al., 2022^36^	Qatar	Cross sectional	2022
Murad et al., 2022^15^	Saudi Arabia	Cross sectional	2022

### 3.1 Healthcare provider‐related determinants

#### 3.1.1 Technical care

This represents healthcare providers' practice concerning their competency, ethics, and confidentiality.^10^ Four studies indicated that overall technical care could contribute to patients' level of satisfaction with healthcare services provided in PC settings.^20^, ^25^, ^29^, ^33^ The ability of a health provider to discuss health problems (including psychological aspects),^20^, ^28^, ^29^, ^30^, ^35^ how to take medications correctly,^22^, ^36^, ^37^ and advice to promote healthy living^13^, ^29^, ^30^, ^37^ were seen as important competencies related to increasing PS with the services provided.

PS was also shown to be affected by the qualification of a PC provider, as the satisfaction scores were more positive towards family physicians compared to general practitioners.^13^, ^15^, ^20^, ^37^ This was found to be significantly related to the fact that visiting a family physician allowed the patient to follow up with the same doctor.^20^, ^28^ Other studies showed that PS was significantly affected when visiting consultant physicians and those with nonsurgical specialties.^23^, ^38^ Patients also favored having specialized doctors in their primary centers.^19^, ^23^, ^39^ The comprehensiveness of the services provided to patients, including full physical examination and accurate and up‐to‐date medical history, were also reported as important elements of PS.^14^, ^24^, ^34^

#### 3.1.2 Interpersonal care

This is usually reflected through proper communication, patient‐centered decision‐making, and interpersonal style.^40^ Good Communication skills were significantly related to high PS scores.^13^, ^20^, ^22^, ^23^, ^24^, ^26^, ^27^, ^29^, ^30^, ^31^, ^32^, ^34^, ^36^, ^38^, ^39^, ^41^, ^42^ Patients' trust in the PC health provider to discuss private psychological health issues was found to positively affect their level of satisfaction.^28^, ^30^, ^33^ On the contrary, attributes related to interpersonal styles, such as respectfulness, humaneness, and friendliness, were frequently reported as essential attributes of PS.^13^, ^14^, ^20^, ^22^, ^25^, ^30^, ^34^, ^37^, ^39^, ^42^

#### 3.1.3 Physical environment

The physical environment was also reported to affect PS. Cleanliness of PC centers,^25^, ^44^, ^46^ comfortable design and layout,^36^, ^46^ enough space in reception and waiting area and their locations within the center,^22^, ^29^, ^46^ availability of air ventilation and air conditioning,^29^ comfortable furniture in waiting area,^39^, ^46^ and availability of public toilets^46^ were reported as factors that may enhance patients' experience in health facilities and improve their level of satisfaction with health services.

Differences between rural and urban regions were found to be significant concerning the cleanliness and design of the PC centers. A study showed that patients in rural regions were significantly more likely to state that the PC was not very clean than those from urban regions.^13^ Moreover, patients in urban regions were likelier to report that it is very easy to get around and within the PC center than patients in rural regions.^13^

#### 3.1.4 Access (accessibility, availability, and affordability)

Access to healthcare is concerned with helping people command appropriate healthcare resources to preserve or improve their health.^43^ It was found that accessibility, availability, and affordability are the main determinants for access to health care that may affect PS.^10^

Accessibility to PC centers was seen as related to PS.^27^ This was found to be related to the convenient location of health centers,^14^, ^15^, ^22^, ^24^, ^26^, ^34^, ^39^, ^44^ suitability of working hours,^22^, ^34^, ^39^, ^44^ physicians spending enough time with patients to explain their conditions,^20^, ^32^, ^33^, ^37^, ^39^, ^42^ shorter waiting time.^13^, ^15^, ^22^, ^23^, ^24^, ^29^, ^32^, ^34^, ^36^, ^45^ Furthermore, one study has shown that implementing a triage system significantly reduces waiting time and increases the level of PS.^16^

However, another study showed that access and utilization of PC services significantly varied between urban and rural areas in relation to opening days/hours and distance.^13^ It was found that respondents from urban regions were significantly more likely to want the center to open early mornings, with those from rural regions most likely to want the center to open for extra days; whereas the distance was significantly more likely to present a problem to those residing in rural regions compared to those from urban regions.^13^ Moreover, patients in urban regions were likelier to report that it is very easy to get around and within the PC center than patients in rural regions.^13^

The availability of health services and the sufficient number of healthcare staff were important determinants of PS.^10^ Patients were more satisfied when there was an adequate number of clinical staff, including lab and pharmacy staff.^41^ Availability of specialized services^22^ and other services such as laboratory tests, the follow‐up appointment system, and referral to the hospital, if needed, were an enhancer of PS in different studies.^29^, ^30^, ^44^ Moreover, the availability of medicines and adequate pharmacy supplies increased PS.^22^, ^29^, ^32^

The affordability of healthcare services and their relation to PS was found to be contradictory in some studies. One study reported that paying for prescribed medicines was not significantly related to PS as patients have not had to pay for medicines.^13^ Another study found that overall satisfaction ratings were significantly related to the type of PC (public or private).^42^

#### 3.1.5 Organizational characteristics

This is related to the operation of services and staff within the PC. Satisfaction with the appointment system was reported in different studies with an improvement in appointment booking after Electronic Medical Records (EMR) implementation.^34^, ^47^ Implementation of EMR was also shown to improve other determinants such as technical care (e.g., physician performance), access (e.g., waiting time), interpersonal care (e.g., communication), continuity (e.g., seeing physician of choice), and outcome (e.g., medical care).^21^, ^47^, ^48^ Walk‐in clinics also showed a high satisfaction rate; however, patients favored having an appointment system run concurrently with the existing walk‐in system.^49^

Integrating PC with hospitals improved PS and experience.^50^ The study showed that, after the integration of PC with local hospitals, there was a significant improvement in satisfaction levels in every functional domain, such as overcrowding at the registration counters, overall improvement in PC cleanliness, improved overall general behavior of the staff, and promptness of services.^50^

With regards to staff operation, on the contrary, satisfaction was seen as high with laboratory, pharmacy, radiology, and dental staff and services as well as with physicians,^29^ while seen as not significantly related to receptionists' communication.^31^

#### 3.1.6 Continuity

Continuity of care could be determined by services such as seeing and following up with the same doctor, referral to a hospital, and accessing hospital records from the PC centers.^14^, ^22^, ^27^, ^34^, ^51^ Patients were more likely to express the desire to consult the same physician, especially if they have chronic conditions such as hypertension, diabetes, asthma,^35^ or psychological problems.^51^ A study indicated that relational continuity (where the care provided by one or more named healthcare professionals with whom the patient can develop a therapeutic and interpersonal relationship) improved the patients' medical conditions and the quality of services.^35^

#### 3.1.7 Efficacy/outcome of care

None of the retrieved studies investigated PS concerning outcomes for care except one.^21^ Patients were seen as satisfied with the outcome of care after the implementation of the EMR system, which was related to adherence to treatment, resolution of symptoms, and improvement of functional status.^21^

### 3.2 Patient‐related characteristics results section

Most patient‐related factors were discussed in the included papers; however, religion, length of stay, and personality factors were not discussed. Nationality was added to the race theme, as mentioned by some studies.

#### 3.2.1 Age

Most of the older population were satisfied,^21^, ^26^, ^27^, ^30^, ^32^, ^36^, ^38^, ^41^ which they reported for physicians' rapport,^38^ pharmacy services,^36^ and the implementation of EMR.^21^ However, a study showed dissatisfaction with physicians' services.^29^ Regarding younger age groups, they were dissatisfied with physicians' services,^30^ consultations,^33^ and pharmacy services^36^; and they were satisfied with opening hours, available appointment times,^18^ and waiting times.^45^

#### 3.2.2 Gender

Several studies demonstrated gender differences in satisfaction.^15^, ^18^, ^19^, ^24^, ^25^, ^26^, ^27^, ^28^, ^29^, ^34^, ^36^, ^37^, ^41^ Women were more satisfied^18^, ^26^, ^27^, ^28^, ^29^, ^34^, ^37^, ^41^ with the general practice clinic in comparison to family medicine,^37^ receptionists services, opening hours, ability to see a doctor, phone communication, other communication aspects,^18^ physicians consultations,^28^ and physicians services.^29^ On the contrary, men were more satisfied in a few studies,^19^, ^25^, ^36^ with the diabetes clinic^19^ and pharmacy services.^36^

#### 3.2.3 Education

As per education, several studies linked satisfaction to education level.^21^, ^25^, ^26^, ^27^, ^29^, ^30^, ^33^, ^34^, ^35^, ^36^, ^38^, ^41^, ^45^ Some studies linked higher satisfaction with PC services^34^ and recognized the importance of continuity of care.^35^ Others associated higher education with low satisfaction with general services,^21^ consultation length,^33^ and pharmacy service,^36^ and they were less likely to receive continuity of care, resolve their personal and psychological problems and comply with physicians' recommendations.^35^ Other studies associated low education status with higher satisfaction rates for PC services^25^, ^26^, ^27^, ^41^, ^45^ and physician rapport.^38^ On the contrary, those with low education levels were dissatisfied with general services^34^ and physician services.^29^, ^30^

#### 3.2.4 Socio‐economic status

Several studies discussed the association of different socio‐economic status aspects with satisfaction.^26^, ^29^, ^30^, ^31^, ^33^, ^34^, ^37^, ^41^, ^45^ Numerous studies discussed satisfaction among high‐income participants,^26^, ^29^, ^30^, ^33^, ^37^, ^41^, ^45^ in which some showed satisfaction with physicians' services^29^, ^30^ and consultations,^33^ and one showed dissatisfaction with general services.^26^ Mixed evidence was shown in the association between low income and satisfaction, where studies showed that those with low income showed high satisfaction in the services^26^, ^41^ and low satisfaction in physicians consultations^33^ and physician services.^30^

There was also mixed evidence regarding the association between employment status and satisfaction. Some studies showed that unskilled laborers had high satisfaction rates in general^26^, ^31^ and physician services.^30^ In addition, one study showed that the unemployed had high satisfaction in general services,^41^ and another showed low satisfaction rates in physician services.^29^ Only one study demonstrated that retired or not working participants have high satisfaction rates.^34^ Students were more dissatisfied with the services in general^31^, ^34^ and physician services,^30^ and only one study showed they were satisfied.^26^

#### 3.2.5 Marital status and race/nationality

Numerous studies explored the association of satisfaction with marital status.^15^, ^25^, ^26^, ^29^, ^31^, ^34^, ^41^, ^45^ Higher satisfaction was seen more among married in the general services^31^, ^34^ in waiting times^45^ and physician services.^29^ Other studies also demonstrated high satisfaction rates among divorced,^25^, ^26^ single,^41^ and widowed participants.^25^ Three studies mentioned the effect of nationality on satisfaction.^15^, ^36^, ^39^ One study showed that satisfaction is higher among nationals,^39^ and another showed that satisfaction is also higher among nationals, but the difference was not statistically significant.^36^

#### 3.2.6 Geographic characteristics

The geographic location was discussed, with the location being rural or urban, and the location or proximity of the PC center to the participants' homes and mixed evidence was found. Both urban and rural residents had concerns about different views and needs regarding the accessibility to the centers.^13^ Other studies showed a difference in satisfaction according to the center's location,^13^, ^26^, ^28^, ^30^, ^34^, ^50^ where some linked longer distances between patients' residences and the center with low satisfaction.^13^, ^26^, ^28^, ^30^ As mentioned earlier, differences in satisfaction were seen between rural and urban regions concerning cleanliness, getting around the center, and working hours.^13^

#### 3.2.7 Visit regularity, health status, and expectations

Only one study discussed visit regularity, in which satisfaction was inversely related to the number of visits to the center and its pharmacy.^36^ Satisfaction was also linked to individual health status. Good health was associated with higher satisfaction^39^, ^42^, ^46^; however, one study associated having a chronic illness with high satisfaction.^38^ In addition, other studies linked satisfaction with personal or family medical history.^26^, ^42^ Only one study associated satisfaction with individual satisfaction with life.^39^ Only one study discussed the patients' expectations, showing that PC physicians knew about them and worked hard to achieve them.^35^

## 4 DISCUSSION

Although some studies recommended improving, strengthening and investing in the PC facilities in the GCC,^52^, ^53^ this review showed the satisfaction of the population with PC services, which can be explained by the development witnessed in healthcare systems in the GCC in recent years on different aspects.^54^, ^55^, ^56^ Furthermore, this review showed the importance of technical factors as a determinant of satisfaction with PC services, which is similar to the literature that showed an association of satisfaction with the time spent with the physician,^57^, ^58^, ^59^ confidentiality,^59^ and comprehensiveness of care,^59^ and other technical factors.^10^, ^60^, ^61^ Moreover, as in other studies,^58^, ^59^, ^60^, ^61^, ^62^ communication skills, respectfulness, humaneness, and trust in the care provider was linked with satisfaction. Other healthcare provider factors, such as physical environment, accessibility, availability, organizational specifications, and continuity of care, were found to affect satisfaction, which were also found as determinant factors of satisfaction in the literature.^57^, ^58^, ^60^, ^61^, ^62^, ^63^, ^64^, ^65^

Regarding patient‐related characteristics, this review found that being old and female is linked to higher satisfaction, which is also similar to the evidence in the literature.^57^, ^58^, ^62^, ^66^ These results can be explained by the fact that the older population have greater time than the younger population to receive health services^67^ and the differences in care received by the older population.^68^ The gender differences can also be explained by the differences in the expectations between males and females about the quality of services provided^69^ and that females tend to use health services more frequently than males.^70^

Although this review showed that being married is linked to higher satisfaction, there is mixed evidence on the effect of marriage, which might be explained by the cultural and social differences between the population in GCC and the western countries. This review showed that students had higher satisfaction rates, which adds to the contradictory evidence about the effect of education.^10^, ^57^, ^60^ As in other studies,^10^ the high‐income class in this review was associated with higher satisfaction. Mixed evidence was found on the effect of educational factors, which is different from the literature as it associated a positive association between education level and satisfaction,^10^, ^57^ which can be explained by the small number of results that evaluated the association in this review.^13^ This review found mixed evidence on the effect of geographic location on satisfaction, in terms of being rural or urban and how far the center is from the population residents, which is also similar to other studies as some in the literature.^10^, ^71^

The evidence showed that the frequency of visits, poor health status or having chronic diseases, matching patients' expectations, and good care outcome is associated with higher satisfaction^10^, ^58^, ^62^, ^66^; however, this review found a lack of evidence regarding their effect on satisfaction as few studies discussed the above‐mentioned factors. Surprisingly few studies examined the effect of nationality on satisfaction, despite the high number of non‐nationals among the GCC populations.^72^

This review has several strengths, to our knowledge this is the first review that examines the PS with PC services in the GCC. Furthermore, it showed a lack of evidence in specific satisfaction‐related areas, such as the relation with nationality, health status, and care outcome. On the contrary, this review has some limitations; as with any review, there is a chance of missing some data, especially since the gray literature was not reviewed, and the search was limited to the year 2000 and after. Almost all the included studies were cross sectional, which can affect the causality relation between satisfaction and the determinants.

## 5 CONCLUSION

This is the first scoping review that examined the nature and range of studies investigating the satisfaction of PC services and the factors influencing it in the GCC. Despite the increasing number of articles examining PS in PC services in GCC over years, this number is relatively low given the importance of satisfaction on health outcomes. The review showed that satisfaction with PC services in GCC is affected by multiple factors related to the healthcare provider and the patients. The review demonstrated that the effects of nationality, health status, and care outcomes on patient satisfaction were not examined calling for future research to examine these areas.

## FUNDING INFORMATION

The authors report no external funding source for this study.

## CONFLICT OF INTEREST STATEMENT

The authors declare no conflict of interest.
